# The association between outpatient follow-up visits and all-cause non-elective 30-day readmissions: A retrospective observational cohort study

**DOI:** 10.1371/journal.pone.0200691

**Published:** 2018-07-17

**Authors:** Liping Tong, Tim Arnold, Jie Yang, Xinyong Tian, Cole Erdmann, Tina Esposito

**Affiliations:** 1 Advocate Health Care, Downers Grove, IL, United States of America; 2 Cerner Corporation, North Kansas City, MO, United States of America; 3 University of Illinois at Chicago, Department of Mathematics, Statistics, and Computer Science, Chicago, IL, United States of America; Illumina Inc, UNITED STATES

## Abstract

**Background:**

As an effort to reduce hospital readmissions, early follow-up visits were recommended by the Society of Hospital Medicine. However, published literature on the effect of follow-up visits is limited with mixed conclusions. Our goal here is to fully explore the relationship between follow-up visits and the all-cause non-elective 30-day readmission rate (RR) after adjusting for confounders.

**Methods and results:**

To conduct this retrospective observational study, we extracted data for 55,378 adult inpatients from Advocate Health Care, a large, multi-hospital system serving a diverse population in a major metropolitan area. These patients were discharged to Home or Home with Home Health services between June 1, 2013 and April 30, 2015. Our findings from time-dependent Cox proportional hazard models showed that follow-up visits were significantly associated with a reduced RR (adjusted hazard ratio: 0.86; 95% CI: 0.82–0.91), but in a complicated way because the interaction between follow-up visits and a readmission risk score was significant with p-value < 0.001. Our analysis using logistic models on an adjusted data set confirmed the above findings with the following additional results. First, time matter. Follow-up visits within 2 days were associated with the greatest reduction in RR (adjusted odds ratio: 0.72; 95% CI: 0.63–0.83). Visits beyond 2 days were also associated with a reduction in RR, but the strength of the effect decreased as the time between discharge and follow-up visit increased. Second, the strength of such association varied for patients with different readmission risk scores. Patients with a risk score of 0.113, high but not extremely high risk, had the greatest reduction in RR from follow-up visits. Patients with an extremely high risk score (> 0.334) saw no RR reduction from follow-up visits. Third, a patient was much more likely to have a 2-day follow-up visit if that visit was scheduled before the patient was discharged from the hospital (30% versus < 5%).

**Conclusions:**

Follow-up visits are associated with a reduction in readmission risk. The timing of follow-up visits can be important: beyond two days, the earlier, the better. The effect of follow-up visits is more significant for patients with a high but not extremely high risk of readmission.

## Introduction

Reducing hospital readmissions remains a significant challenge for many healthcare systems. In a study among Medicare patients by Jencks et al in 2009, about one in five discharged patients were re-hospitalized within 30 days and only 10% of these re-hospitalizations were planned [1]. Hospitals with excessive 30-day readmission rates among patients with acute myocardial infarction (AMI), heart failure (HF), pneumonia (PN), and chronic obstructive pulmonary disease (COPD) have suffered financial penalties through the Hospital Readmission Reduction Program (HRRP) enforced by the Centers for Medicare and Medicaid Services (CMS) [2].

Early follow-up visits are recommended by the Society of Hospital Medicine for patients discharged home—a finding based on expert opinion rather than published studies [3]. However, literature on the effect of follow-up visits to reduce readmissions is limited with mixed conclusions. Several papers based on patients with specific conditions, as well as general hospitalized patients, conclude that early follow-up visits are helpful in preventing 30-day readmissions [3–9]. Other articles show that patients do not meaningfully benefit from follow-up visits [10–12]. These mixed conclusions may reflect the truth or might be due to insufficient adjustment for potential confounding factors or the incorrect choice of methods when comparing the groups with and without follow-up visits.

As pointed out in [7], when the follow-up visit is treated as a fixed indicator with a binary outcome of yes or no, there is a potential bias resulting from the fact that patients who are readmitted earlier will not have a chance for a follow-up visit and hence are included in the “no follow-up” group [7]. This makes the “no follow-up” group seem worse and thus overestimates the benefit of a follow-up visit. To correct such bias, [7] proposed using the follow-up visit as a time-dependent covariate in Cox proportional hazard models. We show in the Results section that this is helpful in addressing the above concern of overestimation. However, it cannot readily answer the question of when a follow-up visit is the most effective. In addition, the proportional hazard assumption can be hard to validate when time-dependent covariates and interaction terms are both significant in the model. Thus, we propose a simple strategy to carefully define the groups with and without follow-up visits and apply traditional logistic models in the analysis so that the above concerns can be addressed. We show that the results from the sample-adjusted logistic models are amazingly consistent with those from survival models.

## Methods

### Data sources

We extracted the clinical and claims data of inpatients from eight Advocate Health Care hospitals located in the Chicago metropolitan area. The cohort included adult (age > = 18) inpatient encounters with discharge dates from June 1, 2013 to April 30, 2015. In addition, for each inpatient encounter the first post-discharge visit information was extracted up to December 31, 2015. Inpatients with a hospital service of Hospice, Obstetrics, Pediatrics, IP Pediatric Rehab, Psychiatric, Inpatient Rehab, or Skilled Nursing were excluded. If patients expired during hospitalization, we excluded the last encounter only. We initially had a data set of 99,660 encounters for 62,940 unique patients. Since patients discharged to a skilled nursing facility, inpatient rehab facility, or long-term care facility are actively monitored by healthcare professionals, a conventional follow-up visit does not serve the same purpose as it does for patients who go home after discharge. Therefore, we focused only on patients discharged to Home (self-care) or Home Health (visiting nurse), which left us with a sample of 66,400 encounters for 46,866 unique patients. Finally, we excluded inactive patients who were defined as those that didn’t have contact with any of the Advocate hospitals within 6 months after discharge from the hospital. These patients most likely switched to other hospitals or expired at home. Including such patients can underestimate the readmission rate in the control group and thus underestimate the treatment effect. We finally ended up with 55,378 encounters for 38,068 unique patients.

This study has been approved by the Advocate Health Care IRB. All data were fully anonymized and the IRB has waived the requirement for informed consent.

### Response variable

A readmission is defined as a non-elective re-hospitalization within 30 days after discharge from the hospital. In the original dataset, there were 11,864 readmissions among 99,660 index admissions, resulting in a readmission rate (RR) of 11.90%. For the final data set, there were 7,217 readmissions out of 55,378 index admissions, resulting in a RR of 13.03%.

### Follow-up visits

Outpatient follow-up visits have been recommended to reduce patients’ readmission risk by the Society of Hospital Medicine. There are two types of follow-up visits considered in current literature: an actual follow-up visit (AFV) and a scheduled follow-up visit (SFV). An AFV is a healthcare provider-patient encounter with encounter type labeled as appointment or outpatient that occurred after the patient was discharged from the hospital while an SFV refers to an encounter that was scheduled but which may or may not have happened.

We focused on the analysis of AFVs. Most of the AFVs occurred in physician offices. However, we did include other types of outpatient encounters such as rehab services, imaging procedures, chemotherapy treatments and so on. We chose not to differentiate between different aspects of these inpatient visits (e.g. medical, surgical, primary diagnosis, etc.) because we are more interested in seeing the general pattern of association between readmission events and outpatient visits in all types of inpatients. Our data on SFVs was restricted to high-risk patients only, which limited a direct analysis on the association between a visit and readmission risk for the general inpatient population. Therefore, assuming AFVs are effective, we were more interested in seeing how AFVs were affected by SFVs.

### Confounding factors

It is important to include potential confounding factors in the model to minimize bias. The factors we included in this study were raw readmission risk score, acute myocardial infarction (AMI), heart failure (HF), chronic obstructive pulmonary disease (COPD) and pneumonia (PN). The raw risk score is the predicted readmission probability, which was developed by the Advocate Cerner Collaborative team [13]. The raw risk score is considered a representative measure of the actual readmission risk of a patient. A raw risk score of 0.068 or less is considered low risk. A score between 0.068 and 0.10 indicates moderate risk and a score above 0.10 is considered high risk. We also included the indicator variables for HF, AMI, COPD and PN in the model because patients with such conditions are more likely to receive other types of interventions such as transitional visits, heart failure clinic visits, extra education, and so on. By including these factors in the study, we hope to minimize confounding effects so that we might draw a more reliable conclusion.

### Inter-correlation among inpatient encounters of the same patient

An inpatient encounter is an admission event, which might be a readmission to another inpatient encounter in the dataset. To deal with the correlations among inpatient encounters of the same patient, the method of deduplication [13] or generalized estimating equations (GEE) [14] can be applied. However, it was shown that for the purpose of predicting 30-day readmission risk, when a sample size is large enough, neither de-duplication nor GEE gains more precision over the basic models that simply ignore correlations [15]. Therefore, we decided to ignore such correlations and to use the basic models for analysis in this study.

### Hospital diversity

Our data was extracted from eight Advocate hospitals in the Chicago area with patients across a wide spectrum of cultural, ethnic and socioeconomic circumstances. Besides the standard discharge protocol of readmission prevention education for high risk patients, several additional hospital-specific programs were also available in these hospitals to overcome barriers in language, culture and other factors. Considering such diversity, we can apply the random effect models in both survival and logistic analysis to examine the inter-hospital variation. Since results for the regular and random effect models in both survival and logistic analysis are very similar, we included only results from regular models in the Results section for simplicity. Fitting parameters on random effect models were listed in Tables A and B in S1 File.

### Definition of the groups with and without follow-up visits

As discussed in Introduction, the survival models with a time dependent covariate can sufficiently take into consideration the time-dependent feature of both events of readmission and intervention so that a reliable assessment can be made. However, to answer the question of when is the most effective time for a follow-up visit, we need to be more creative.

Our strategy is to first define a threshold *S_E_* for an early follow-up visit. Then we compare differences in readmission rates between the yes-intervention and -no-intervention groups for different values of *S_E_* and choose the one with the greatest difference. In published literature, *S_E_* can vary from 5 days to 2 weeks. We allow *S_E_* to vary from 1 to 29 days so that we can search for an optimal choice in the space of all possibilities.

The definitions of the yes-intervention and no-intervention groups are not as trivial as they seem due to the time-dependence feature for both the readmission and follow-up events. We describe our strategy in the following. For each fixed value of *S_E_*, we first exclude encounters readmitted on or before day *S_E_*, regardless of whether or not they have a follow-up visit before the readmission. What is left in the sample are encounters at risk of readmission after day *S_E_*. Encounters in the sample with an actual follow-up visit on or before day *S_E_* are included in the yes-intervention group. Encounters without an actual follow-up visit on or before day 30 are included in the no-intervention group. Encounters with an actual follow-up visit after day *S_E_* but before day 30 are not included in either the yes-intervention or no-intervention group because there can be a partial effect from a later intervention. Including them in either of the groups can result in biased conclusions. With such defined groups, we can then compare readmission rates and apply logistic models to explore the association between an intervention and readmission risk.

### Statistical analysis

We focused our analysis on AFVs. We first applied the Cox proportional hazard model to evaluate the overall association between a follow-up visit and a readmission event. As in Sharma et al (2010), to avoid time-dependent bias, a follow-up visit must be treated as a time dependent covariate. That is, patients are in the “no follow-up visit” group until they have their first follow-up visit. The proportional hazard assumption for each covariate was examined by plotting the log of cumulative hazard rates against time. The readmission event was censored by 30 days and the follow-up event was censored by 29 days.

We then used the properly defined yes- and no-intervention groups to compare the difference of readmission rates for different values of *S_E_* and to find out the optimal time for an AFV. Next, with *S_E_* being fixed at the optimal value, we applied multivariate logistic models adjusting for risk score, HF, AMI, COPD and PN, to find out the featured groups that might benefit the most from follow-up visits. Analyses were performed with R 3.2.2 and SAS 9.4.

## Results

### Characteristics of the study population

Out of the 55,378 active inpatient encounters discharged to Home or Home Health, 26,436 (47.74%) had at least one follow-up visit within 30 days. There were 1,929 readmissions within 30 days in these patients, which generated a readmission rate of 7.30%. There were 28,942 (52.26%) inpatient encounters without any follow-up visit within 30 days of discharge. The number of readmissions in these patients was 5,288 which resulted in a readmission rate of 18.27%. Direct comparison of these two readmission rates was not reliable due to concerns of potential bias.

We first applied the Cox proportional hazard model in the analysis. Table 1 lists the basic characteristics of the study population and the estimated hazard ratios for potential confounding factors. The cells in columns 2 (Yes) and 3 (No) are bold if the p-value to test the difference is less than 0.05, where p-values were obtained using two-sided t-tests. The cell in column 3 is bold with stars if the hazard ratio is different from 1 at the significant level of 0.05. The interaction between follow-up visit and raw risk score was significant. Therefore, the hazard ratio for raw risk score was not listed and the effect will be explained later.

**Table 1:** Characteristics of patients with and without follow-up visits for survival models (n = 55,378).

	Follow-up Visit within 30 Days		
Variable	Yes (n = 26,436)	No (n = 28,942)	Estimated HR (95% CI)
Raw Risk Score, mean (SD)	**0.12 (0.09)**	**0.15 (0.11)**	**-**^a^
AMI_Current, No. (%)	601 (2.27)	615 (2.12)	0.96 (0.81, 1.14)
AMI_Historical, No. (%)	2516 (9.52)	2627 (9.08)	0.96 (0.89, 1.04)
HF_Current, No. (%)	**2146 (8.12)**	**2594 (8.96)**	0.79 (0.73, 0.85) ***^b^
HF_Historical, No. (%)	**8241 (31.17)**	**9861 (34.07)**	1.15 (1.08, 1.21) *** ^b^
COPD_Current, No. (%)	**1754 (6.63)**	**2183 (7.54)**	0.96 (0.88, 1.05)
COPD_Historical, No. (%)	**7285 (27.56)**	**9140 (31.58)**	1.04 (0.98, 1.10)
PN_Current, No. (%)	1098 (4.15)	1137 (3.93)	0.82 (0.73, 0.92) *** ^b^
PN_Historical, No. (%)	**4556(17.23)**	**5540 (19.14)**	1.18 (1.12, 1.26) *** ^b^

^a^The estimated HR is not available for Raw Risk Score due to the interaction between AFV and Raw Risk Score.

^b^Cells with stars in this column indicate that the estimated hazard ratios are significantly different from 1 (p ≤ 0.05).

Based on the data shown in Table 1, we concluded that patients with follow-up visits had lower raw risk scores, lower proportion of heart failure (current or historical), lower proportion of COPD (current or historical), and lower proportion of historical pneumonia. Patients with current heart failure or pneumonia had lower hazard of readmissions comparing to those without. However, patients with historical heart failure or pneumonia had higher hazard of readmissions comparing to those without.

### Results from Cox proportional hazard models

**Fig 1:**
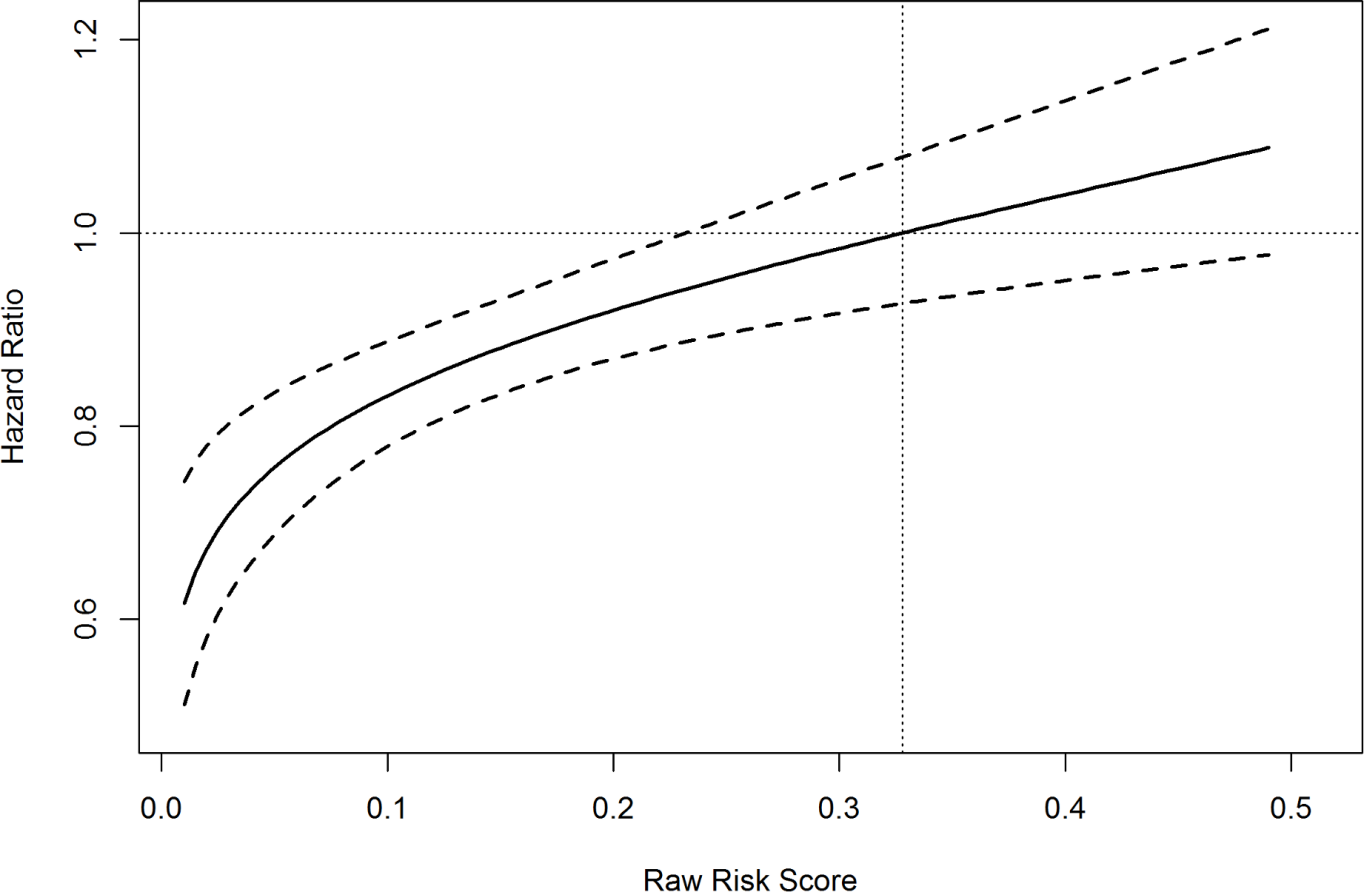
Estimated hazard ratio for a follow-up visit for patients with different risk scores.

The interaction between AFV and raw risk score was significant (p < 0.001). The hazard ratio for the logit of raw risk score was 2.28 (95% CI: 2.16, 2.41) with a follow-up visit and 2.01 (95% CI: 1.95, 2.08) without a follow-up visit. This is consistent with our expectation that a higher raw risk score yields a higher hazard of readmission. In addition, Fig 1 shows the hazard ratios for a follow-up visit at different values of raw risk scores. The hazard ratio approached 1 when the raw risk score increased to 0.328, which indicated that follow-up visits were associated with lower readmission risk for most patients except those with extremely high raw risk scores. In our data, about 8.6% encounters had raw risk scores above 0.3. We applied the Cox proportional hazard model to the subset of data with raw risk scores above 0.3. The estimated hazard ratio for follow-up visits was 0.91 (95% CI: 0.81, 1.04). This indicated that follow-up visits alone might not work well for these patients at extremely high risk of readmission.

It is of interest to know that in the proportional hazard model that included all confounding factors but excluded interactions, the estimated hazard ratio of yes vs no for follow-up visit on the risk of readmission is 0.89, with a 95% confidence interval (0.84, 0.94). This indicated that on average follow-up visits were significantly associated with a reduction of readmission risk. This is consistent with the results by Sharma et al in 2010 although they were using Medicare data for COPD patients, where they concluded that patients who had a follow-up visit had a significantly reduced risk of readmission with a hazard ratio of 0.91.

### Most effective time for a follow-up visit

**Fig 2:**
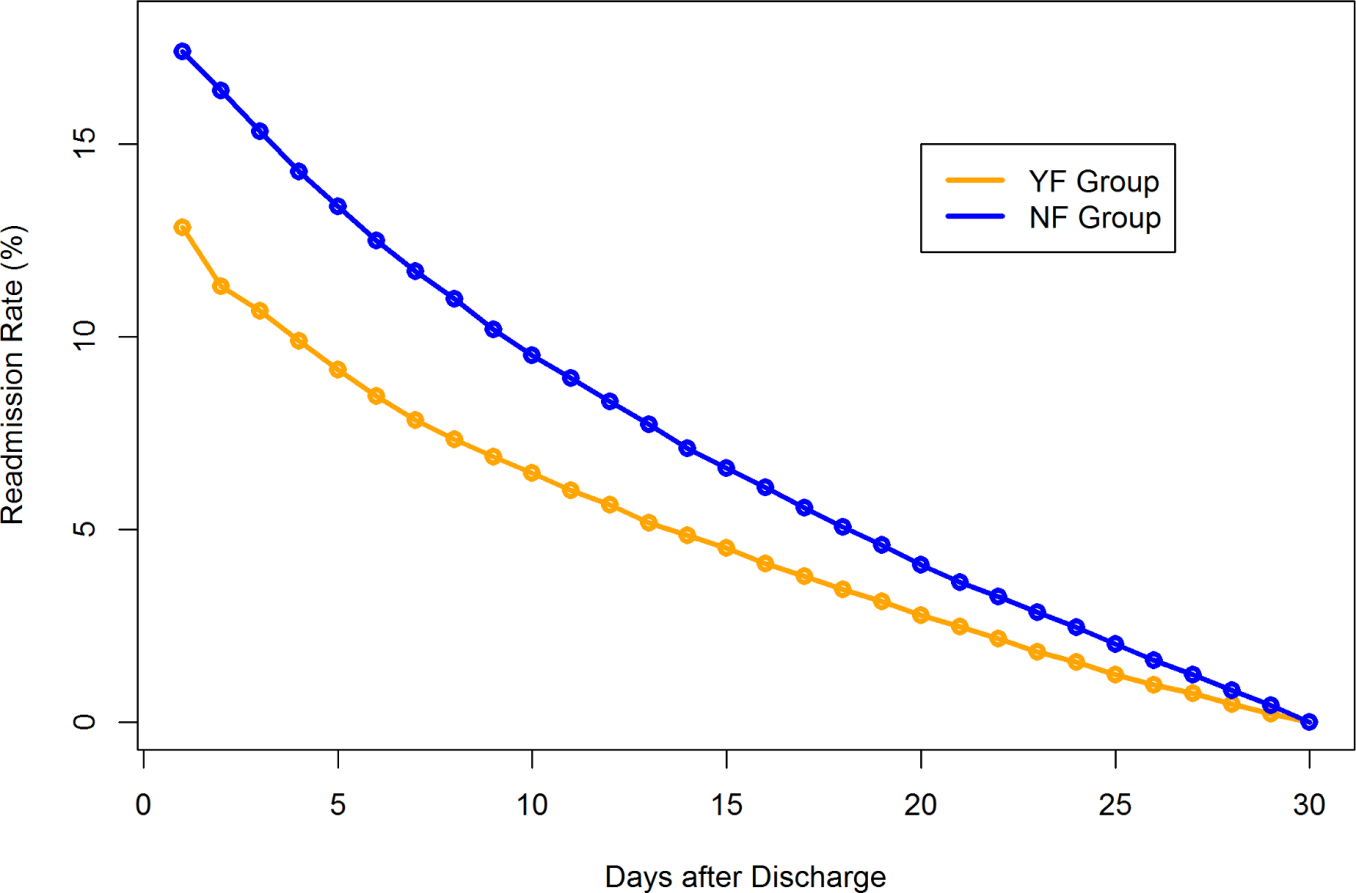
Comparison of Readmission Rate (RR) for Patients with (YF) and without (NF) follow-up visits on or before various days.

Due to varying expectations on readmissions at different time points, we cannot directly compare the readmission rates for patients with follow-up visits. However, we can generate comparable groups with (YF) and without (NF) early follow-ups and compare the difference of RR between these two groups. To that end, we calculated the readmission rate of patients with and without follow-up visits from day 1 through day 29. The results are displayed in Fig 2. We concluded that a follow-up visit within 2 days was associated with the greatest reduction in RR. Such association beyond two days was still significant, but the strength of it diminished over time.

Next, we focused on patients with 2-day actual follow-up visits (YF group, n = 2,412) versus those without (NF group, n = 28,290) for further analysis. It turned out that the RR in the YF group was 11.32% as compared to 16.39% in the NF group, which generated an (unadjusted) odds ratio of 0.65 (95% CI: 0.57–0.74). The adjusted odds ratio (with confounding factors, but without interactions) was 0.72 (95% CI: 0.63–0.83). Table 2 presents the basic characteristics for the groups with (YF) and without (NF) early follow-ups. The YF group had lower risk scores on average and a lower proportion of patients with historical COPD.

**Table 2:** Characteristics of patients in follow up visit and no follow up visit groups for logistic models (n = 30,702).

	Early Follow-up Visit		
Variable	Yes (n = 2,412)	No (n = 28,290)	Estimated OR (95% CI)
Raw Risk Score, mean (SD)	0.13 (0.10)	0.15 (0.11)	**-**^a^
AMI_Current, No. (%)	51 (2.11)	603 (2.13)	0.98 (0.78, 1.25)
AMI_Historical, No. (%)	228 (9.45)	2547 (9.00)	0.94 (0.84, 1.05)
HF_Current, No. (%)	231 (9.58)	2524 (8.92)	0.76 (0.68, 0.85) ***^b^
HF_Historical, No. (%)	825 (34.20)	9576 (33.85)	1.18 (1.09, 1.28) ***^b^
COPD_Current, No. (%)	165 (6.84)	2119 (7.49)	0.92 (0.82, 1.04)
COPD_Historical, No. (%)	**684 (28.36)**	**8888 (31.42)**	1.03 (0.96, 1.11)
PN_Current, No. (%)	87 (3.61)	1102 (3.90)	0.82 (0.70, 0.97) ***^b^
PN_Historical, No. (%)	426 (17.66)	5375 (19.00)	1.26 (1.16, 1.36) ***^b^

^a^The estimated OR is not available for Raw Risk Score due to the interaction between AFV and Raw Risk Score.

^b^Cells with stars in this column indicate that the estimated odds ratios are significantly different from 1 (p ≤ 0.05).

### Results from logistic models

**Fig 3:**
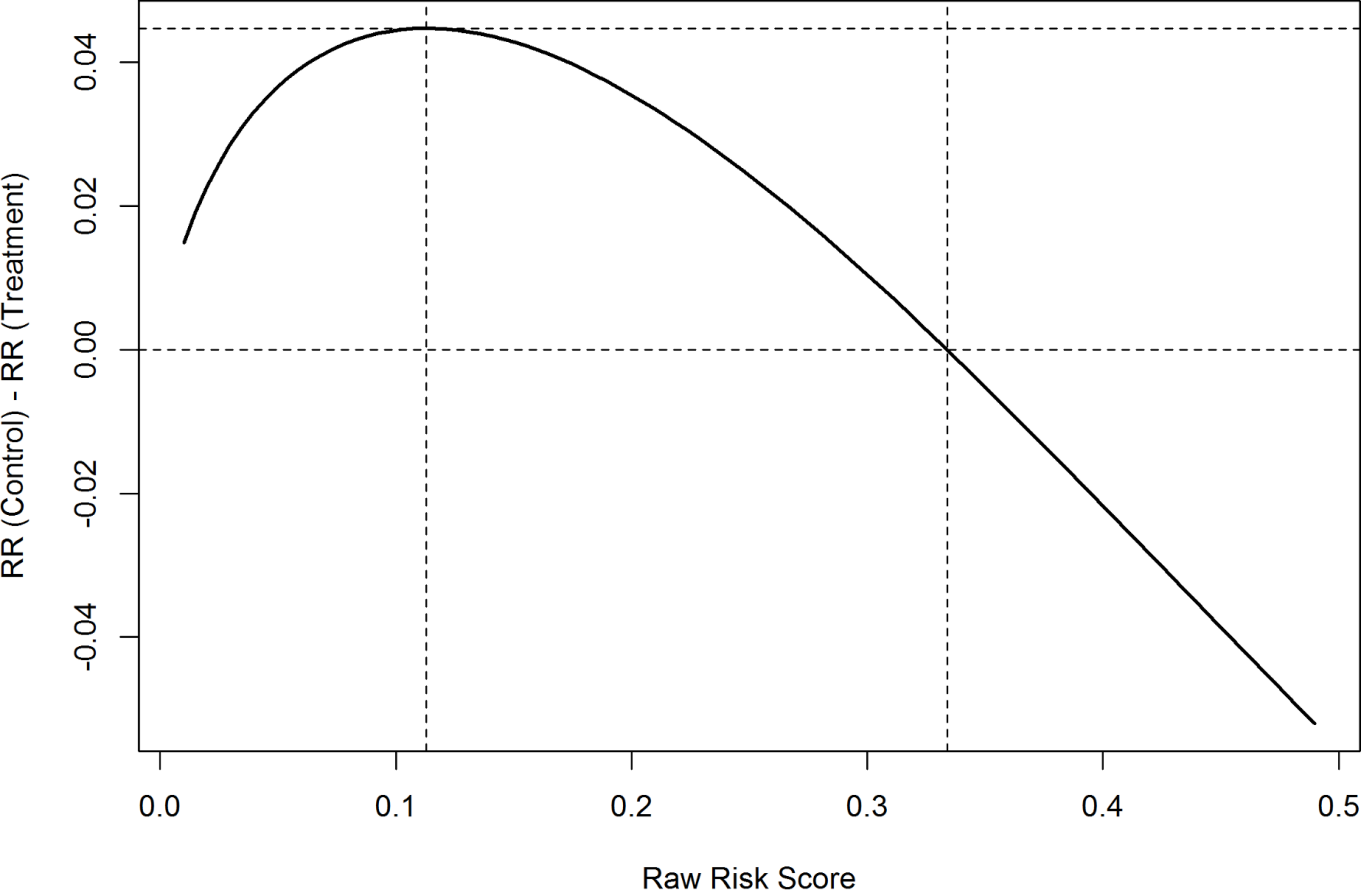
The effect of early follow-up visits on readmission risk for patients with different raw risk scores.

The last column in Table 2 shows the estimated odds ratio for each risk factor. We drew very similar conclusions when comparing to results from the proportional hazard models. That is, a higher raw risk score always predicted a higher probability of readmission. Patients with current heart failure or pneumonia had a lower readmission risk, while patients with historical heart failure or pneumonia had a higher readmission risk. Again, the interaction between follow-up visits and raw risk scores was significant. To further explain the model, we generated Fig 3 using the logistic model that included only follow-up visits, raw risk scores and the interaction between them. Fig 3 shows the effect of follow-up visits on the readmission risk for patients with different risk scores. It turned out that the effect was maximized when the raw risk score equaled 0.113. Patients with a risk score of 0.113 were considered high but not extremely high risk patients for readmission. This effect decreased as the readmission probability got further from 0.113 in either direction, and it approached 0 when the raw risk score equaled 0.334. Patients with a raw risk score of 0.334 or above were considered extremely high risk patients. Many of them had complicated conditions as well as frequent visits to the hospital. It was consistent with our experience that a single follow-up visit might not be effective in reducing readmissions for these patients. However, it did not mean that AFV would do harm to such patients. In addition, since this optimal value was sensitive to the coefficient of the interaction term, we calculated the 95% confidence interval for this coefficient and obtained optimal values with the coefficient being at the margin values. We then had 0.056 at the lower boundary and 0.146 at the upper boundary, which can be viewed as the approximate 95% confidence interval for the optimal value of the raw risk score of patients to maximize the reduction in readmission risk. In our data, there were about 45% of the encounters with raw risk scores between 0.056 and 0.146.

### Scheduled follow-up visit

We used the 2-day actual follow-up visit as a threshold and checked the relationship between the actual and scheduled follow-up visits. In the 66,400 encounters discharged to Home or Home Health, we further excluded 175 encounters with an appointment date prior to discharge or greater than one year after the discharge date, as well as 686 encounters readmitted within 2 days. We ended up with a data set containing 65,539 encounters. The p-value to test independence between AFV and SFV was less than 0.001, which indicated a strong association between these two variables.

Table 3 shows the joint distribution of actual and scheduled follow-up visits. We saw that of the patients scheduled to visit a doctor within 2 days, about 30% did visit a doctor within 2 days. Patients without such a scheduled visit saw a doctor within 2 days less than 5% of the time. This indicated that scheduling 2-day follow-up visits before discharging patients to home or home health was associated with a lower readmission risk.

**Table 3:** Joint distribution of the actual and scheduled follow-up visits (n = 65,539).

		Scheduled Follow-up Visits (Day)							
		< = 2		>2 and < = 30		>30		No SFV	
		No.	%	No.	%	No.	%	No.	%
Actual Follow-up Visits (Day)	< = 2	469	**30.06**	523	**4.59**	7	**2.33**	2,400	**4.59**
	>2 and < = 30	509	32.63	6,508	57.08	112	37.21	20,847	39.88
	>30	316	20.26	2,352	20.63	135	44.85	16,731	32.00
	No AFV	266	17.05	2,018	17.70	47	15.61	12,299	23.53
Total		1,560		11,401		301		52,277	

## Discussion

In this paper, we have explored the association between follow-up visits and the risk of readmission using both proportional hazard models and logistic models. In the proportional hazard models, it is critical to treat follow-up visits as a time-dependent variable. Otherwise, the effect can be overestimated, which leads to misleading conclusions. In the logistic models, it is critical to first specify a threshold to define an early follow-up visit event and then exclude patients no longer at risk as well as patients with later follow-up visits.

Both analyses concluded that timely follow-up visits for patients discharged to Home or Home Health was strongly associated with a lower risk of readmission. Follow-up visits can work differently for patients at different levels of readmission risk. Such association was the strongest for patients with high but not extremely high raw risk scores. Patients with a current condition of heart failure or pneumonia had a lower risk of readmission compared to those without, which might be due to other successful intervention programs. However, patients with a historical condition of heart failure or pneumonia had a higher risk of readmission compared to those without. This indicates that intervention programs on these patients might also be considered to further reduce readmissions.

In addition, we found out that a follow-up visit within 2 days worked best for patients with a raw risk score of 0.113. A patient was much more likely to have a 2-day follow-up visit if that visit was scheduled before the patient was discharged from the hospital. However, we understand that in practice, it might be a challenge to have patients come back within 2 days. Our results showed that the best strategy is to aim for follow-up visits within 2 days. If not, patients can still benefit from follow-up visits—especially those which occur as soon after 2 days as possible.

The major confounding factor, the raw risk score, is not necessarily available in other medical systems. However, similar scores, either automatically generated from an EMR [16–20] or manually calculated using algorithms such as LACE [21], can be easily obtained. Therefore, our conclusions on the complicated association between follow-up visits and readmissions can be readily double checked and possibly generalized to other health care providers to improve medical practice.

### Limitations

This study has several limitations. First, it is an observational study. Although we have tried our best to adjust for potential confounding factors, there are always other possible explanations on the association between follow-up visits and reduced readmissions because of unmeasured variables such as socioeconomic status (SES), medical literacy level, medication adherence and so on. For example, patients with timely follow-ups might have higher SES, might care more about their health and might be more willing to comply with medications and to follow professional medical suggestions. The observational study only reveals an association between follow-up visits and readmissions. The causal effect conclusion can be drawn only through a controlled experiment that minimizes bias, such as the study in [22].

It is a challenge to properly exclude inactive patients from the data. The ideal way would be keeping contact with each of the patients discharged home and obtaining timely information when a patient dies or switches to another hospital so that the readmission rates for both groups with and without early follow-up visits can be estimated more accurately. However, it is very hard to keep track of every patient in practice. Our method to exclude patients without any hospital contact for six months after discharge to home is a compromise, which might be problematic for smaller hospitals with high turnover rates of patients.

The readmission rate of 13% was much lower than the RR of 19.6% as in [1] for three possible reasons: (1) patients in our sample were younger ((≥ 18) than Medicare patients (≥ 65); (2) the overall RR has become lower over time since the enforcement of HRRP began in 2012 [23]; (3) information on readmissions could be missing if patients received care at other hospitals, which could result in an underestimation of the actual RR. Since we are generally missing more information for patients with whom we have no further contact, this might result in an underestimation of the association between follow-up visits and readmission events. Our effort to define active patients is not the ideal way to solve this problem, but it can correct such an underestimation to a certain degree.

The strategy we proposed for logistic models is easy to follow and generates consistent results with survival analysis. Nevertheless, it has obvious limitations. We lose power by discarding a large proportion of data. Also, by excluding patients with later follow-up visits from the data, the estimation on readmission rate is no longer accurate. We intend to show in a future paper that such a method is appropriate under certain model assumptions but can be problematic under others. It is methodologically feasible to use all the data and make a direct fair comparison for patients with follow-up visits to those without by developing new models and testing statistics, but that is beyond the scope of this paper and is worth exploring further.

## Supporting information

S1 File Table A. Raw results of the time dependent Cox proportional hazard model, both regular and random effect, including all factors, using data with a sample size being 55,378. Table B. Raw results of the logistic model, both regular and random effect, including all factors, using data with a sample size being 30,702.
(DOCX)

S1 Data File (CSV)
