# Supplementary material for: The association between outpatient follow-up visits and all-cause non-elective 30-day readmissions: A retrospective observational cohort study
Source: PLoS One. 2018 Jul 17;13(7):e0200691. doi: 10.1371/journal.pone.0200691 (PMC6049937; doi:10.1371/journal.pone.0200691)
Supplement: S1 File — Table A. Raw results of the time dependent Cox proportional hazard model, both regular and random effect, including all factors, using data with a sample size being 55,378. Table B. Raw results of the logistic model, both regular and random effect, including all factors, using data with a sample size being 30,702. (DOCX) [file pone.0200691.s001.docx]

**Supporting Information**

We have included the raw fitting results of the time dependent Cox proportional hazard model in Table A and the logistic model in Table B. For the purpose of comparison, we run both models with a random effect for hospital as well. The results with a random effect are very similar to those without.

| **Table A:** Raw results of the time dependent Cox proportional hazard model, both regular and random effect, including all factors, using data with a sample size being 55,378 | | | | | | |
| --- | --- | --- | --- | --- | --- | --- |
| n = 55,378 | Time Dependent Cox Proportional Hazard Model | | | Time Dependent Cox Proportional Hazard Model with a Random Effect for Hospital | | |
| Parameter | Parameter  Estimate | P-value | Hazard  Ratio | Parameter  Estimate | P-value | Hazard  Ratio |
| Follow up Visit | 0.08951 | 0.1091 | . | 0.10364 | 0.0646 | . |
| Logit of Raw Risk Score | 0.70014 | <.0001 | . | 0.69458 | <.0001 | . |
| Follow up Visit * Logit of Raw Risk Score | 0.12481 | <.0001 | . | 0.12821 | <.0001 | . |
| AMI_cur | -0.03856 | 0.6608 | 0.962 | -0.03891 | 0.6583 | 0.962 |
| AMI_his | -0.0404 | 0.3249 | 0.960 | -0.04284 | 0.2971 | 0.958 |
| Heart_Failure_cur | -0.2425 | <.0001 | 0.785 | -0.24114 | <.0001 | 0.786 |
| Heart_Failure_his | 0.13739 | <.0001 | 1.147 | 0.13553 | <.0001 | 1.145 |
| COPD_cur | -0.04049 | 0.3604 | 0.96 | -0.03952 | 0.3723 | 0.961 |
| COPD_his | 0.03627 | 0.2038 | 1.037 | 0.03681 | 0.1989 | 1.037 |
| Pneumonia_cur | -0.19498 | 0.0009 | 0.823 | -0.19398 | 0.001 | 0.824 |
| Pneumonia_his | 0.16828 | <.0001 | 1.183 | 0.17156 | <.0001 | 1.187 |

| **Table B:** Raw results of the logistic model, both regular and random effect, including all factors, using data with a sample size being 30,702 | | | | | | |
| --- | --- | --- | --- | --- | --- | --- |
| n = 30,702 | Logistic Model | | | Logistic Model with a Random Effect for Hospital | | |
| Parameter | Parameter  Estimate | P-value | Odds  Ratio | Parameter  Estimate | P-value | Odds  Ratio |
| Intercept | -0.3342 | <.0001 | . | -0.3659 | 0.001 | . |
| Follow up Visit | 0.2138 | 0.1812 | . | 0.2128 | 0.1841 | . |
| Logit of Raw Risk Score | 0.7576 | <.0001 | . | 0.7511 | <.0001 | . |
| Follow up Visit * Logit of Raw Risk Score | 0.3179 | 0.0003 | . | 0.3199 | 0.0003 | . |
| AMI_cur | -0.0161 | 0.8947 | 0.984 | -0.00516 | 0.9663 | 0.995 |
| AMI_his | -0.0631 | 0.2854 | 0.939 | -0.06822 | 0.2492 | 0.934 |
| Heart_Failure_cur | -0.2737 | <.0001 | 0.761 | -0.2745 | <.0001 | 0.760 |
| Heart_Failure_his | 0.1659 | <.0001 | 1.181 | 0.1559 | <.0001 | 1.169 |
| COPD_cur | -0.0808 | 0.1941 | 0.922 | -0.0782 | 0.2092 | 0.925 |
| COPD_his | 0.0309 | 0.4303 | 1.031 | 0.04214 | 0.2849 | 1.043 |
| Pneumonia_cur | -0.1941 | 0.0188 | 0.824 | -0.1867 | 0.0243 | 0.830 |
| Pneumonia_his | 0.2272 | <.0001 | 1.255 | 0.2281 | <.0001 | 1.256 |
